# Perspectives About Older Adult and Family Member Engagement in Medication Management Across Transitions of Care

**DOI:** 10.1111/jocn.70405

**Published:** 2026-06-19

**Authors:** Grace Marconi, Maneesh Prasad, Kerry Hwang, Caitlin Deery, Kelly Ottosen, Elizabeth Manias

**Affiliations:** ^1^ Monash Nursing and Midwifery, Monash University Clayton Victoria Australia; ^2^ Monash Health Clayton Victoria Australia

**Keywords:** family engagement, medication management, medication safety, patient engagement, patient participation, qualitative interviews, transitions of care

## Abstract

**Aim:**

To explore perspectives of older patients, family members, and healthcare professionals regarding patient and family engagement in medication management across transitions of care.

**Design:**

A qualitative study utilising semi‐structured interviews with patients aged ≥ 65, family members and healthcare professionals.

**Methods:**

Participants were recruited from four hospital sites from a major public hospital system which services a large socioeconomically diverse population of metropolitan Melbourne. Interviews were analysed using inductive content analysis.

**Results:**

In total, 67 patients, 66 family members, and 69 healthcare professionals participated in the study (*n* = 202). The three themes generated were: challenges in supporting patients and families in communication, factors influencing patient and family ability to communicate, and approaches to enhanced engagement.

**Conclusion:**

There is a need for strategies which empower and encourage patients and family members to engage in medication management, which, despite complex barriers, could be achieved through enhanced communication.

**Implications for Patient Care:**

Proposed strategies present possibilities for older patients and families to engage in medication management across transitions of care, providing opportunities to reduce medication errors and adverse events with the potential to improve patient outcomes.

**Impact:**

This research uncovers the complexities relating to older patient and family engagement and decision‐making, and generates strategies to improve communication in medication management.

**Reporting Method:**

Conducted in accordance with the Consolidated Criteria for Reporting Qualitative Research.

**Patient or Public Contribution:**

This study sought insights from older patients who were admitted to hospital and family members, with experience in managing medications across transitions of care. Two consumers with lived experience as caregivers for older patients and involved in medication management were invited to contribute to manuscript preparation and provided detailed feedback, which was implemented in the final manuscript.

**Trial Registration:**

Australian New Zealand Clinical Trials Registry (ANZCTR) on 24 July 2024: ACTRN12624000901505

## What Does This Paper Contribute to the Wider Global Clinical Community?

- Identifies older patients' and their family members' needs and preferences, in regard to medication management during transitions of care, are often disregarded, with communication between healthcare professionals taking priority.
- Establishes that older patient and family member trust in the expertise of healthcare professionals and a belief of inadequate medication knowledge contribute to passivity and poor engagement in medication management.
- Despite complex barriers, proposed strategies present possibilities for enhanced communication and older patient and family member engagement in medication management across transitions of care.

## 1 Introduction

Medication management is the management of safe, effective and quality medication use with a patient‐centred focus which aims to reduce medication errors (Australian Commission on Safety and Quality in Health Care 2021). In older patients, medication management is complex, largely due to the presence of co‐existing chronic conditions, often requiring ongoing treatment with multiple medications, leading to polypharmacy. Polypharmacy is defined as the regular use of five or more medications by a patient (Masnoon et al. 2017). Globally, polypharmacy is increasingly prevalent due to the growth of an ageing population with a recent review estimating that approximately 45% of individuals aged 65 and older worldwide are affected (Kim et al. 2024). Polypharmacy can be either appropriate or inappropriate, with the latter including the prescription of potentially inappropriate or unnecessary medications (Masnoon et al. 2017). Although polypharmacy may be necessary and is not avoidable in all cases, it has been identified as a major contributor to medication‐related problems leading to harm in older patients, due to the increased complexity of medication regimens (Lee et al. 2023).

Transitions of care involve movements of patients within or between healthcare settings, and include consultations with different healthcare providers (Australian Commission on Safety and Quality in Health Care 2025b). These are high‐risk periods with more than 50% of medication errors occurring at these times (Australian Commission on Safety and Quality in Health Care 2025b). Medication errors have the potential to cause medication‐related harm, which poses a significant burden on healthcare systems globally. A systematic review by the World Health Organization estimated that medication‐related harm affected 1 in 20 patients globally, with over a quarter of this harm being classified as severe or life threatening (World Health Organization 2023). Economic implications of preventable patient harm are profound, with estimates from the Organisation for Economic Co‐operation and Development indicating that in developed countries, adverse events account for approximately 15% of total hospital expenditure (Slawomirski and Klazinga 2022). To reduce the occurrence of such harm, several health organisations, including the National Institute for Health and Care Excellence, the Australian Commission on Safety and Quality in Healthcare, the Institute of Healthcare Improvement and the World Health Organization, have all advocated for a greater focus on accurate information transfer during transitions of care (Almanasreh et al. 2016).

A major driver of medication errors in older patients is ineffective communication (Manias et al. 2021; Ozavci, Bucknall, Jorm, et al. 2021). Observational studies have shown that older people often encounter difficulties in communicating about their medications as they move across different healthcare settings, and despite having a desire to be involved, many lacked the necessary skills needed to do so effectively (Manias et al. 2016; Ozavci, Bucknall, Woodward‐Kron, et al. 2021). Past studies have also indicated that older patients often feel disempowered in making medication‐related decisions, with language, cultural, cognitive and disease‐related barriers further affecting their ability to participate (Manias et al. 2019; Ozavci, Bucknall, Jorm, et al. 2021; Tobiano et al. 2019). This lack of engagement increases the likelihood of medication‐related errors occurring, which has the potential to contribute towards adverse events and unplanned hospital readmissions (Becker et al. 2021; Kemp et al. 2017).

Patient engagement is defined by the World Health Organization as ‘the process of building the capacity of patients, families, carers, as well as health care providers, to facilitate and support the active involvement of patients in their own care, in order to enhance safety, quality and people‐centredness of health care service delivery’ (World Health Organization 2016). The Australian Commission on Safety and Quality in Health Care (ACSQHC), through its *Partnering with Consumers* standard, emphasises the importance of engaging consumers in the delivery of safe and high‐quality health care (Australian Commission on Safety and Quality in Health Care 2025a).

Past studies have highlighted the importance of engaging older patients and family members in medication management across transitions of care; however, these studies focus primarily on medication changes in hospital or power relations (Ozavci, Bucknall, Woodward‐Kron, et al. 2021; Manias et al. 2024). While these studies demonstrate that a lack of engagement persists, there remains a need to further explore patient and family engagement in medication management by considering patients and families of diverse demographic characteristics and backgrounds, and their perspectives of what helps and hinders possibilities for engagement (Manias et al. 2024, 2019).

## 2 The Study

### 2.1 Aims

The aim of this study was to explore the perspectives of older patients, family members, and healthcare professionals regarding patient and family engagement in medication management within hospital settings and across transitions of care. Additionally, this study aimed to determine factors that affect engagement in medication management and decision‐making and to identify possible strategies that patients, family members and healthcare professionals consider would be effective in enhancing engagement.

## 3 Methods

### 3.1 Design

This is an exploratory qualitative research study which utilises semi‐structured interviews to gain an understanding of participants' experiences and opinions around medication management across transitions of care.

### 3.2 Study Setting and Recruitment

Older patients, family members and healthcare professionals were recruited from four public hospitals within a major metropolitan health system in Victoria, Australia. The study sites included general medical, surgical and geriatric rehabilitation wards which service a large and diverse socioeconomic area.

Recruitment was through a combination of purposive and convenience sampling. Purposive sampling was used to ensure that the sample included participants of different ages, types of admitted wards for patients, and professional backgrounds for healthcare professionals. Convenience sampling was used as the researchers approached people who were available on the ward on a particular day. Researchers confirmed patient eligibility through consultation with the nurse‐in‐charge or bedside nurse, and their suitability to participate in the study was discussed. Eligible patients or visiting family members were then approached at the bedside by a researcher who provided the participant information sheet and consent form to the patient or family member, along with an informal information session about key aspects of the project. It was not necessary for patients and families to be recruited as a dyad.

Recruitment of healthcare professionals occurred through information sessions conducted with the healthcare team during handover periods or by a researcher approaching staff on the ward. One of the researchers provided a copy of the participant information sheet and consent form to healthcare professionals and provided verbal details about the project. All participants were able to ask questions and clarify any issues before deciding to participate and were given up to 2 days to consider participation. Participants were viewed as experts of their lived experience, with researchers seeking to understand the participants' perspectives.

### 3.3 Inclusion Criteria

Inclusion criteria for older patients were hospitalised individuals aged 65 years or older who had at least one prescribed medication. This was based on the definition of older adults by the Australian Institute of Health and Welfare (Australian Institute of Health and Welfare 2024). Participation from individuals who are of culturally and linguistically diverse (CALD) backgrounds was encouraged and interpreters were available for these participants. Attempts were made to access patients with mild or moderate cognitive impairments to ensure patient views with diverse cognition conditions are sought. Exclusion criteria for patients are those with severe cognitive impairment, severe dementia, and haemodynamic instability, or at end of life. Inclusion criteria for family members were anyone who identified as a relative or friend of an older patient which was identified by researchers asking visitors of eligible patients on the wards. Inclusion criteria for healthcare professionals were qualified nurses, doctors and pharmacists who worked at the study sites. There were no exclusion criteria for family members or healthcare professionals.

### 3.4 Data Collection

Semi‐structured interviews were conducted by the research team between October 2024 and January 2025. The researchers who conducted the interviews (GM, MP, KH, CD, KO) included three research fellows, two of which hold PhDs and the other a registered pharmacist, and two PhD candidates who are a registered pharmacist and nurse. Interviews with patients and/or family members took place at the patient bedside or at a sitting area within the ward and were often conducted together. Interviews were completed this way out of convenience for hospitalised patients; however, researchers were conscious of conducting interviews in multi‐bed bays where other potential participants would be able to hear and attempts were made to minimise this issue. Interviews with healthcare professionals took place on the ward, in staff breakrooms or at a sitting area within the ward, and were conducted either individually or as focus groups of up to three, although due to availability there was one focus group conducted which consisted of seven nurses. To ensure data saturation and that participant insights were adequately represented, a large sample was recruited. This need for adequate representation was due to the heterogeneity in the participant population, including persons from diverse socioeconomic backgrounds and different hospitals and ward types. Interviews lasted between 2.06 and 51.36 min with an average length of 13.30 min.

Interview questions were based on a semi‐structured topic guide developed by the research team based on existing literature and researcher experience (Manias et al. 2024; Ozavci, Bucknall, Woodward‐Kron, et al. 2021). The open‐ended questions of this topic guide comprised the following topics: patient and family involvement and their level of understanding of prescribed medications, patient and family involvement in decision‐making about medications in hospital and during transitions of care, and how healthcare professionals assist both patients and family in understanding of medications and medication decision‐making. The questions from the interview topic guide are outlined in Supporting Information S1. Probing questions were also utilised to obtain further depth of participants' perceptions and experiences. While the term ‘engaged’ has been used throughout this article, the term ‘involved’ was used in the topic guide as this was deemed to be more easily understood by participants, but these terms can be used interchangeably in this context.

Professional interpreters were available for patients or family members who did not speak English, but only one interview was conducted with an interpreter. All interviews were audio recorded and later transcribed. Demographic data of participants were collected and managed using REDCap (Research Electronic Data Capture), hosted and managed by Helix (Monash University) (Harris et al. 2019). REDCap is a secure, web‐based software platform designed to support data capture for research studies, which provides an audit trail for validated data capture.

### 3.5 Data Analysis

Audio recordings of interviews were transcribed verbatim using Zoom (Zoom Inc., San Jose, USA), and the transcripts were manually checked by the researchers for accuracy. Interviews were analysed using inductive content analysis, because of its flexibility in analysing both the manifest explicit text and latent text in terms of relationships between the triad of patients, family members and healthcare professionals (Graneheim and Lundman 2004). Data source triangulation, through the inclusion of the three participant groups and diverse locations and ward types, allowed for deeper understanding and validity. Three researchers (GM, MP, KH) immersed themselves in the data by reading the transcripts multiple times. An open coding framework was developed based on notes and thoughts discussed during the immersion process and codes were developed from contextual words and sentences (Graneheim and Lundman 2004). The three researchers coded interviews separately, then through multiple iterations reviewed the coding framework and made changes based on consensus. Once the codes were agreed, they were abstracted into themes and sub‐themes collectively (Elo and Kyngäs 2008). NVivo 15 Collaborative Cloud (QSR International Pty Ltd., 2024) was used to manage and organise all codes and themes.

### 3.6 Ethical Considerations

Ethical approval was obtained through the hospital system's Human Research Ethics Committee and Monash University. A verbal description of the study and what participation would involve was provided to all participants, as well as a written information sheet, and time was given to consider participation. Informed consent was obtained from all participants before interviews were conducted. Participation was voluntary and withdrawal from the study at any time was possible.

### 3.7 Rigour and Reflexivity

The research team comprised persons with expertise in pharmacy, nursing, ageing, gerontology, rehabilitation, consumer engagement, and digital health. Rigour was ensured in multiple ways, as outlined in Table 1.

**TABLE 1:** Rigour of the research process.

Dependability	Enabled by creating an audit trail that guided decision‐making for conducting the study, and regular meetings with the whole research team
Authenticity	Enabled by using illustrative quotations and narratives to support identified themes
Transferability	Facilitated by explaining the research setting where interviews were conducted
Confirmability	Demonstrated by illustrative quotations, and providing information demographics of participants
Credibility	Strengthened by interviewing multiple participant types and across multiple hospitals sites and wards, as well as member checking with steering group committee members who were not involved in the interview or data collection, which consisted of consumers, nurses and a geriatrician
Reflexivity	Enabled by researchers reflecting on their assumptions, background, experiences and values in the conduct of the research

## 4 Findings

### 4.1 Characteristics of Participants

In total, 132 interviews were conducted with 202 individual participants, including 67 patients, 66 family members, and 69 healthcare professionals, across 12 wards at 4 hospital sites. Participants were assigned a unique identification code and all transcripts were de‐identified to protect their privacy. The participant characteristics are outlined in Tables 2, 3, 4, and the number of different interview types is depicted in Table 5.

**TABLE 2:** Patient characteristics.

Patients	67
Age	
Mean	79.5 years
Range	65–92 years
Biological sex	
Female	35
Male	32
Country of birth^a^	
Oceania and Antarctica	44
North‐West Europe	9
Southern and Eastern Europe	7
North Africa and Middle East	3
Americas	2
Southern and Central Asia	1
Sub‐Saharan Africa	1
Main language spoken at home^b^	
English	59
Arabic	2
Spanish	2
Albanian	1
Armenian	1
Croatian	1
Dutch	1
Hindi	1
Italian	1
Macedonian	1
Portuguese	1
Tamil	1
Motor and sensory deficits	38
Hearing loss	20
Vision impairment	15
Mobility issues	14
Use of aids	46
Walker	37
Hearing aid	10
Pillbox	6
Wheelchair	4
Walking stick	2
White cane	1
Admitting health problem^c^	
Fall	22
Renal	7
Gastrointestinal	6
Neurological	6
Respiratory	5
Infection	4
Cancer	3
Delirium/confusion	3
Cardiovascular	1
Musculoskeletal	1
Complications post‐surgery	1
Dislodged drain tube	1
Fluid drainage	1
Foot ulcer	1
Gastrointestinal and cardiovascular	1
General unwellness	1
Not coping at home after recent discharge	1
Number of prescribed medications on day of interview	
Mean	12.28
Range	2–24
Type of ward recruited from	
General medical	33
Geriatric rehabilitation	16
Surgical	11
General medical/Surgical	7

^a^ Grouped according to the Standard Australian Classification of Countries (SACC).

^b^ Some participants selected English and another language.

^c^ Admission data only recorded for 65 of the participants.

**TABLE 3:** Family member characteristics.

Family members	66
Age^a^	
Mean	61.6 years
Range	28–86 years
Biological sex	
Female	54
Male	12
Relationship to patient	
Son or daughter (including in‐laws)	38
Spouse	20
Sibling	4
Cousin	1
Ex‐wife	1
Grandson	1
Niece	1
Living situation	
Lives with patient	28
Does not live with patient	38

^a^ Age data only recorded for 64 of the participants.

**TABLE 4:** Healthcare professional characteristics.

Healthcare professionals	69
Age	
Under 25	11
26–30	22
31–35	10
36–40	13
41–45	8
46–50	4
51–55	0
Over 56	1
Biological sex	
Female	55
Male	14
Discipline group	
Nurse	45
Pharmacist	14
Doctor	10
Years of experience	
Less than 1 year	5
1–2 years	11
3–5 years	17
6–10 years	17
11–15 years	10
16–20 years	8
21–30 years	1

**TABLE 5:** Interview type.

Individual interviews	79
Patient and family member dyad interviews	24
Patient and two family member triad interviews	7
Family member only dyad interviews	4
Healthcare professional group interviews	17

There were three major themes generated from the interviews: challenges in supporting patients and families in communication, factors influencing patient and family ability to communicate, and approaches to enhanced engagement. Figure 1 outlines the themes and sub‐themes.

**FIGURE 1:**
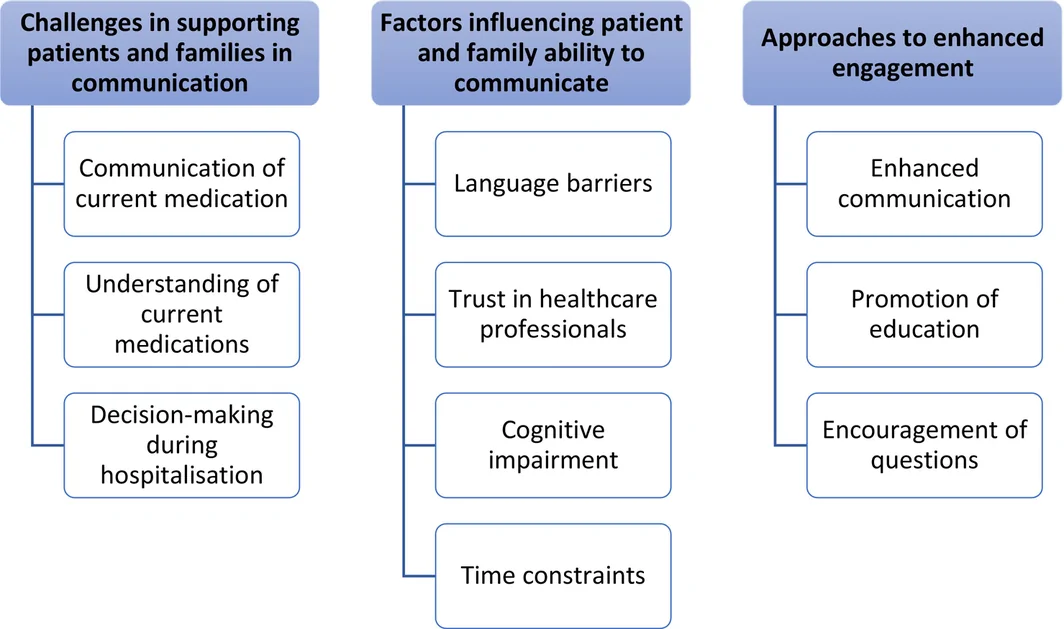
Themes and sub‐themes. [Colour figure can be viewed at wileyonlinelibrary.com]

### 4.2 Themes

#### 4.2.1 Challenges in Supporting Patients and Families in Communication

Patient and family participation and level of engagement in medication management during transitions of care were explored, including issues affecting involvement in decision‐making. There were three sub‐themes identified: communication of current medications, understanding of current medications, and decision‐making during hospitalisation.

##### 4.2.1.1 Communication of Current Medications

Ensuring medication continuity was important for patients and family as they transitioned into the hospital environment, and there were multiple methods in which patients managed, and communicated their current medications to healthcare professionals. Common methods included using either written or electronic lists, taking photos of current medications, and physically bringing medication to hospital. One patient who had written out their own medication list stated: ‘The first thing [health professionals] say is “what medication are you on?”. And I say, “hang on a minute, I'll give you a list”. And I straight away pull out that list’ *(PT001_Male_71)*. In another example, a family member of a patient with limited English proficiency indicated that they would bring their Webster‐pak in to show staff: ‘Yeah, that's been the harder bit because obviously her English is not the best. So, she would just show them the Webster‐pak and that would be it. Just the Webster‐pak and go through what medication she's on at the time’ *(FM186_Daughter_46)*. For patients who were not able to communicate their medications themselves, family members were sometimes relied upon, with one family member explaining: ‘I went through all the meds with [the healthcare professional]… I just know them off by heart because I do her dosette box’ (*FM189_Daughter_57*).

##### 4.2.1.2 Understanding of Current Medications

Patients and families had varied understanding of current medications, both at home and during the hospital stay, with differences around who was responsible and how to access further information. Many patients indicated that they were responsible for their own medications, and were aware of how many medications they were on and what each medication did: ‘No, I do it myself. I've got my box with all my things and look, you just know the colour of the box or the shape of it and things like that. I know them all*’ (PT174_Patient_Female_75)*. Others knew that they took medications but had limited understanding of them, with one patient stating ‘Some of [the medications] that I take, I have no idea what I'm taking them for’ *(PT032_Patient_Male_75)*. Some family members were involved with helping patients manage their medications outside of hospital: ‘…at hospital, definitely, the nurses are looking after it. But yeah, yeah, at home I do. We help with a lot of it’ *(FM038_Daughter_43)*. Other family members indicated that they were not involved in medication management as the patient was independent, which prompted reflection that maybe they should be more involved or aware: ‘I wouldn't know what he takes and what they're for. Which is not very really good. Really, I should know, shouldn't I?*’ (FM132_Spouse_Female_88)*.

##### 4.2.1.3 Decision‐Making During Hospitalisation

Although patients and family often participated by communicating their current medication on admission to hospital, their involvement in medication discussions and decision‐making was limited upon transfer to other hospital settings. For some patients, they were satisfied with limited involvement in decision‐making, particularly if their current medical condition was stable: ‘The way it's working now seems all right, and I'll just go with the flow’ *(PT089_Patient_Male_75)*. Another reason for not wanting to be further involved in medication decisions was that patients and family felt they lacked sufficient medical knowledge compared to healthcare professionals: ‘Well, they know what they're talking about. I'm a lay person, so I don't know’ *(PT205_Patient_Female_86)*.

Patients and family members often mentioned that healthcare professionals made medication decisions and they were only notified of medication changes after the changes were actioned. One daughter explained that medication changes were noticed once the medication list was provided at discharge: ‘Yeah, it's never something that they tell you. Again, the only time I've sort of found out that there's been a change is if we leave the hospital and then a new medication list gets given and then the pharmacist will go through it with you and go, this is a new [medication] tablet. That's the only time I'll really know if there's a change’ (*FM186_Daughter_46*). This situation deprived the patient or family member of the opportunity to be involved as ‘the patient doesn't get told… what is being done medication wise for them, then they have no clue and therefore they cannot be involved in that decision*’ (HP087_Pharmacist)*.

Doctors explained that it was infeasible to involve patients or family in every medication change, as often there could be multiple changes during the day. Due to the multiple changes, these tended to be communicated to patients or family members after the changes were implemented, at the next ward round: ‘I don't think it would be possible to involve them at every single point, because, I mean, we usually only have time to see them usually once a day, whereas decisions about medications change throughout the day, whether it's based off their blood test or their blood pressure or blood sugar, or the results of a scan or something’ *(HP170_Doctor)*. Doctors involved patients or family with symptom‐relieving medication: ‘if it's a symptomatic medication, like pain or nausea that's really guided by the patient… I don't tend to make many decisions there…so I think the symptomatic ones is really heavily guided by the patient*’ (HP171_Doctor)*. Despite this view, healthcare professionals acknowledged that patients could be more engaged with their medication management which would help them to manage any new medications once they left hospital, ‘as the best advocates for them is themselves’ *(HP006_Nurse)*. One healthcare professional stated that they could be more involved in decision‐making by ‘just being aware of what they're taking’ *(HP144_Nurse)*.

Both patients and family members were happy to have family supporting the patient, so long as the autonomy of the older person was respected, with one family member stating ‘you don't want to take away someone's rights… that's [the patients] choice’ *(FM206_Daughter_45)*.

I will always take [FM017's] thoughts on board. However, we don't think the same way, and we will respect each other's rights. (PT016_Patient_Female_70)

Other family members indicated that perhaps they would be more involved if the patient was not able to or struggling themselves: ‘If [PT001] was struggling and I was able to be there with him … just as that support, I think everyone needs a support system there’ *(FM002_Wife_69)*.

#### 4.2.2 Factors Influencing Patient and Family Ability to Communicate

The involvement of patients and family in medication‐related decisions in hospital was largely determined by factors impacting on communication and influenced both the nature and the extent of medication‐related discussions conducted during transitions of care. These identified factors comprised four sub‐themes: language barriers, trust in healthcare professionals, cognitive impairment and time constraints.

##### 4.2.2.1 Language Barriers

The professional interpreter service played an important role in facilitating clear and accurate communication between patients and family members of different linguistic backgrounds and healthcare professionals. Although available 24 h a day, healthcare professionals needed to ‘book it online’ *(HP152_Pharmacist)* and ‘…unless they are somehow onsite, it [was] usually a phone thing’ *(HP087_Pharmacist)*. This need to plan limited the practicality of the service in some clinical contexts: ‘…because often with interpreters, it depends on their availability. You might need them instantly, but they're like, I can come in twenty minutes or so’ *(HP124_Nurse)*. This lack of flexibility was further noted by a family member: ‘…The [interpreter has] only been used for other things like meetings with consent and meetings with doctors about other stuff but not for medication’ *(FM186_Daughter_46)*.

In situations where arranging a professional interpreter was not feasible, healthcare professionals occasionally relied on bilingual staff to help facilitate communication with patients. While acknowledging that this was ‘… really against [hospital] protocol’ *(HP119_Nurse)*, many healthcare professionals saw this as a pragmatic workaround and preferred to rely on a bilingual colleague rather than a family member for interpretation as ‘… maybe the family member might not translate it exactly …but I would trust my colleague’ *(HP092_Nurse)*.

Family members were also often relied upon to help with language interpretation for patients with limited English proficiency during routine clinical interactions such as medication administration, particularly when no colleagues spoke the language

Sometimes I didn't understand. Always, I have my son with me. You can, you know, explain to me …because sometimes the language too fast for me or something. I got my son with me always. He explain [sic] to me. (PT004_Patient_Male_68)

Healthcare professionals often called family members to help explain, particularly during medication administration or if the patient was refusing medication as patients were more receptive to communication from family, with one family member stating ‘if she doesn't understand, they call us… so she knows exactly what they're giving to her’ *(FM172_Daughter_64)*. However, there were occasions when family were not present on the ward and as pointed out by a healthcare professional, ‘…not all families are amenable to us calling them at all hours of the day’ *(HP137_Nurse)*.

Furthermore, use of complex medical terminology by doctors during clinical discussions also impeded on the ability of some patients and their families to engage in conversations about their medications. As described by a patient, ‘Well, when you ask a doctor, most doctors talk doctor language, and I don't understand that’ *(PT089_Patient_Male_75)*. This situation was even more challenging for patients and family members with limited English proficiency: ‘…and if they're struggling with the understanding and because of the language as well, it might feel overwhelming’ *(FM145_Son_57)*. They found it helpful when healthcare professionals communicated in plain and simple language as one family member explained, ‘I say to them sometimes ‐ my English, it's not so very well. Like a small kid in school. You can speak to me like that so I can understand’ *(FM146_Spouse_Female_74)*.

##### 4.2.2.2 Trust in Healthcare Professionals

A high level of trust in healthcare professionals was commonly expressed by both patients and family members: ‘…I trust them and use the hospital and take it for granted that everything's great’ *(PT216_Patient_Male_85)*. Consequently, decisions about medications were often deferred to the healthcare professionals given their extensive training and qualifications

My honest answer would be, I am more than happy with the reputation of the medical people that I am dealing with. They've been fully trained. This is what their life's about all the time. How could I, as a person, say, I think you're doing the wrong thing there? (PT231_Patient_Male_84)

As explained by a family member, inadequate knowledge and understanding about health conditions and medications limited their ability to actively participate in decision‐making: ‘Not being medically trained, you're not going to know. You're taking the doctor's word because they're a doctor’ *(FM041_Son_54)*.

##### 4.2.2.3 Cognitive Impairment

Healthcare professionals perceived family engagement to be limited to instances where the patients were unable to understand or advocate for themselves. Doctors who prescribed medications for older people reflected that such patients were more likely to have delirium or dementia, which affected their ability to engage in their care, and this was a reason for doctors to involve family in decision‐making: ‘…or whether there is some sort of underlying cognitive impairment. It does make it tricky, and that's… where there's a lot of reliance upon family members [or] next of kin’ (*HP210_Doctor*). For patients with dementia, communication challenges were identified to affect their ability to participate in medication management, with patients not being able to communicate their needs and being unable to understand medication information as ‘it just goes over’ *(FM060_Spouse_Female_70)*.

##### 4.2.2.4 Time Constraints

Busy ward environments, competing priorities for healthcare professionals and changing attending staff, at times made patients perceive that their care was disjointed or fragmented: ‘…Quite often you will be talking to them and all of a sudden they get called away….and then you will see a different doctor next time. So, yeah, the continuity is not there’ *(PT192_Patient_Male_73)*. Some patients and families also perceived time limitations during ward rounds as a barrier to engaging in comprehensive discussions regarding medication management: ‘…how many patients he's got to look at and so when he comes around here, he tells us in general terms, what is happening, why we're taking off one medication and we're looking at another and that's about all. So, we don't know the specifics of what is happening’ *(FM088_Spouse_Male_86)*. Workload challenges, including patient load and the fast‐paced dynamic environment, made it difficult for healthcare professionals to co‐ordinate timing during ward rounds to discuss medications as observed by a family member: ‘…the pharmacist will come and explain what it's for and whatnot, but then sometimes it's a little bit too late, because the doctors have already kind of prescribed it…’ *(FM206_Daughter_45)*.

#### 4.2.3 Approaches to Enhanced Engagement

Patients and family members referred to possible approaches that could be used to improve their engagement in medication management and decision‐making across transitions of care. The strategies and approaches identified comprised three sub‐themes: enhanced communication, promotion of education, and encouragement of questions.

##### 4.2.3.1 Enhanced Communication

Many identified approaches aimed to enhance bidirectional communication between patients, family members, and healthcare professionals, as well as interprofessional communication throughout transitions of care. Family members and healthcare professionals reported having experienced concerns with correctly and efficiently reconciling the patients' current medications on admission. It was therefore suggested ‘that everybody has an actual medication list’ *(FM206_Daughter_45)* so that ‘there's a way of having an availability of medications that the patient is on that's easily accessible’ *(FM071_Daughter_63)*. Approaches regularly identified by family members and healthcare professionals included patients carrying a medication list on their person, sharing current medication lists with family, using medication list phone applications, and accessing the national digital health record, known as My Health Record.

With patients often feeling overwhelmed by the amount of information given, all participant groups proposed that healthcare professionals should give patients ‘a bit more time to digest the information…, and then checking in later… [because] there's a lot of information that's given all at once… but questions don't occur until like half an hour later’ *(FM134_Daughter_33)*. To further mitigate this *‘information overload’ (HP209_Pharmacist)* and to improve understanding of medications, it was suggested ‘to actually start explaining the medications prior to discharge, not only when they're going home. There's lots of medications… and it kind of goes like whoosh’ *(HP013_Nurse)*. Furthermore, as some patients were not able to manage on their own or were unable to relay information onto family, many participants expressed importance ‘that a family member is present’ (*FM189_Daughter_57*) during ward rounds and discharge, and that ‘they should tell the person that is taking [the patient] home what's going on, … because [the patients] don't really take it all in’ *(FM011_Spouse_Female_81)*. Updates for family was a common strategy mentioned, with one family member suggesting ‘just a quick update on the phone, just tell us that we have started this because of this… then we'll know and be reassured that this is what's happening’ *(FM081_Sibling_Female_68)*. A family member also proposed that ‘family should be invited and told how [the hospital] works, because if you haven't been in this hospital system before, or experienced anything of this nature, … you just don't know what happens’ *(FM153_Daughter_62)*.

Family members and healthcare professionals suggested that there should be more proactive communication between the hospital staff and the patient's general practitioner, local pharmacist, or primary care specialist, in order to avoid potential medication errors due to a lack of shared information. One family member reflected that ‘when you are leaving the hospital and you get all your information with the medication, sometimes that medication information doesn't go to the [general practitioner] properly… then they use their own discretion with some medications without sort of connecting with the hospital’ *(FM187_Daughter_52)*.

When medication information was not communicated by hospital staff directly to external healthcare providers or family, there was a reliance on the patient to relay this information themselves. To further mitigate potential medication errors, reduce confusion and improve understanding of medications, written communication was suggested as a helpful approach and ‘in [the patients] preferred written language as well’ *(FM134_Daughter_33)*. Current communication about medication during the hospital stay was largely verbal, with the only consistent written communication being the discharge summaries given before leaving, which prompted patients and family to suggest written communication during the hospital stay as well.

Some sort of written something because people forget, things have got weird names and weird stuff, so I think something written. I think they almost could have little slips of paper that say we're giving you this for whatever, so that they can then relay it to families. (FM031_Daughter_60)

Patient friendly discharge summaries were seen to be ‘a really useful tool for them to… have that information… in front of them in simple terms’ *(HP210_Doctor)*. When a medication information resource was suggested, many patients, family members and healthcare professionals agreed that it ‘would be very helpful’ *(HP211_Doctor)*, with one healthcare professional mentioning that they have ‘brochures for lots of things… but there is nothing for medications…, it would be like information for them, so they know I can ask my doctor if I have some issues with my medication’ *(HP062_Nurse)*. Furthermore, to enhance communication, family members suggested that ‘there might be a way to use technology to help get information across faster’ *(FM188_Daughter_54)* and to allow family to access information such as ‘an online … login sort of thing where… [the family can see] this is what [the patient is] on and then have a list of [medications]’ *(FM186_Daughter_46)*.

##### 4.2.3.2 Promotion of Education

Education was identified as a key approach to improve patient and family engagement and understanding of medications across transitions of care. Healthcare professionals and family members identified that pharmacists were integral to providing in‐depth education about medications and that ‘they explain how to take it, when to take it, when to stop it, …[and] what needs to be monitored’ *(HP015_Nurse)*. While pharmacist education was typically conveyed during discharge counselling, all groups suggested that healthcare professionals can further improve understanding through educating ‘the average person on what the medications are, what they do, and pros and cons’ *(FM041_Son_54)* throughout the hospital stay, such as ‘when [nurses] administer medications, [they should] tell them… this medication is for this one and this one for that one’ *(HP014_Nurse)*. This continued education was regarded as important as ‘if the patients understand what they're taking and why they're taking it, then they're more likely to be compliant … [and it] helps them with their own health journey’ *(HP136_Nurse)*.

Teach‐back was a suggested approach to promote medication understanding, with one healthcare professional saying that ‘you can… reinforce what you've already said to them, and then use teach‐back, and see if they can understand the seriousness of … why they need to take it the prescribed way’ *(HP026_Pharmacist)*. Many healthcare professionals mentioned medication initiation and ‘self‐administration trials… [to] make sure they can do it when they are discharged’ *(HP123_Nurse)*.

##### 4.2.3.3 Encouragement of Questions

To improve patient and family understanding of medications, a frequently proposed approach was to encourage questioning

Just knowing about what medications they're taking and asking for education on what the medications are being given… to give them more information is by being asked questions. (HP006_Nurse)

Healthcare professionals suggested that by ‘having the pharmacist introduce themselves early… is quite important… [so] patients will know… that [the pharmacist is] the one to ask questions to’ *(HP025_pharmacist)*. Furthermore, ‘ongoing education of families and patients, that they're encouraged to ask questions and making them feel a part of the situation’ *(HP176_Nurse)*, gives them opportunities to ask and improves their engagement in medication management.

A question prompt resource was suggested with ‘questions they can ask to the [healthcare professionals] … [that] they can refer to’ *(HP122_Nurse)* and was said to be a useful way to give ‘both the patient and the family tools to empower them and speak up and ask those questions’ *(HP210_Doctor)*. All groups mentioned the usefulness of this resource, with one doctor indicating it would be a ‘sheet to prompt them in case there's anything that they didn't know that they didn't know’ *(HP170_Doctor)* and a patient stating that they would ‘probably ask questions where [they] probably would not have asked questions [previously]’ *(PT089_Patient_Male_75)*.

## 5 Discussion

This study provides new insights into the perspectives of patients, family members, and healthcare professionals on the engagement of patients and family in medication management during transitions of care. Communication between healthcare professionals was often prioritised over patient and family member needs, preferences and values in regard to medication management. Over‐trusting the expertise of healthcare professionals to the point where passivity ensued and a belief of inadequate medication knowledge contributed to poor engagement in medication management. Despite these concerns, possibilities for enhanced communication were identified in the presence of complex constraints.

Technical medication information, primarily communicated verbally, was often overwhelming to patients. As a result, patients expressed a need for more time to process information, desired having family present or wanted written information. Nevertheless, there was a disconnect between what the patients and family wanted and what occurred in practice. Jargon‐laden medication information was prioritised during rushed ward rounds, with healthcare professionals only seeking family involvement if the patient was cognitively impaired. This prioritisation indicates that patients' needs were not being met and healthcare professionals took a reactive approach to communication. Medication understanding and patient outcomes have been shown to improve through tailored communication which takes patient preferences into consideration (Ozavci, Bucknall, Jorm, et al. 2021), with family participation in discussions leading to improved communication with healthcare professionals (Rotman‐Pikielny et al. 2007). While ward rounds provide an opportunity for family members to participate, these are haphazardly organised due to time pressures and healthcare professionals' responsibilities across various wards, making it difficult for family to attend (Manias et al. 2019; Bassetti et al. 2025; Manias 2015). Furthermore, our findings are supported by past research which shows that a difficulty understanding medical explanations meant patients desired family support during ward rounds (Rotman‐Pikielny et al. 2007); however, healthcare professionals are unlikely to actively involve family due to workload constraints (Manias 2015). This is despite the vital role of family in medication decision‐making, with a lack of involvement resulting in an absence of shared decision‐making and inadequate medication understanding (Manias et al. 2019; Manias 2015).

Patients and family members expressed they had inadequate medical knowledge to enable involvement in decision‐making and hence they put their trust in the expertise of healthcare professionals. This view suggests that patients and family emphasised scientific understanding of clinical symptoms and treatments to manage health conditions, and devalued their own expert knowledge of their needs, preferences and values. Patients and family members did not appear to recognise themselves as legitimate and valued contributors to decision‐making, with perceived inadequacy and lack of confidence discouraging them from asking questions and offering input, resulting in an over‐reliance on healthcare professionals. Previous research has highlighted the significance of empowerment in promoting a patient's ability to make informed decisions and confidence to participate in discussions, and the importance of providing opportunities for participation in patient‐centred care (Hickmann et al. 2022). However, current organisational structures contribute to maintaining the hierarchical positions of medical dominance, which further devalues the perspectives of patients and family members and limits patient‐centred care (Grover et al. 2022). Therefore, strategies which empower patients and family members to recognise the importance of their perspectives and their role in patient‐centred care may be beneficial to increase engagement in medication management and participation in decision‐making, ultimately improving health outcomes and reducing medication errors and adverse events (Becker et al. 2021; Kemp et al. 2017; National Aging Research Institute 2006).

There were multiple factors identified which caused communication breakdowns, including language barriers, cognitive impairment, and time constraints; however, the findings highlight the potential for enhanced communication in the presence of these complex constraints. While many barriers to effective communication stem from systemic issues, such as the impracticality of interpreter services and workload pressures, effective approaches indicate the value of multimodal communication strategies and the critical role of family in medication management. In particular, tailored information delivery and education, through verbal, written, and active communication, have been shown to enhance medication comprehension and patient‐centred care, ultimately improving patient outcomes and readmission rates (Ozavci, Bucknall, Jorm, et al. 2021; Rameshkumar et al. 2022). Existing literature has demonstrated that patients and family members lack confidence in asking questions, as they do not feel that they have the knowledge or are unsure of what to ask, affecting their ability to participate in decision‐making (Stoll et al. 2025; Manias 2015). A suggested approach was to provide patients and family with a question prompt resource that would empower them through knowing the right questions to ask. By asking the questions important to them, healthcare professionals could provide tailored, effective, and time‐efficient active education, improving comprehension and the ability to relay information on to family or other healthcare professionals. Previous research has demonstrated that this approach of empowering patients and family with questions to ask improves engagement, communication, and safe medication use (Herold et al. 2020). Through being easy to understand and translate, and by improving the efficiency of communication with healthcare professionals, this approach could address communication issues due to language barriers, cognitive impairment and time constraints. Furthermore, a lack of medication knowledge by patients and family can cause challenges and delays in achieving accurate medication reconciliation on admission, which is important in addressing polypharmacy and is a critical time‐point for medication errors and potential harm (Stark et al. 2020; Sutanto 2025). Another suggested approach to improve communication was an up‐to‐date patient‐held medication list that is shared with family. This strategy increases participation in medication management, despite a patient's ability to verbally communicate or recall medications, allowing more accurate and efficient information transfer during transitions of care, which is demonstrated to improve medication reconciliation and reduce incidences of medication errors (Stark et al. 2020; Almanasreh et al. 2016).

Past research has identified that miscommunication is the leading cause of issues with medication management across transitions of care and demonstrates that this can be mitigated through interventions targeting communication which are shown to improve patient outcomes and reduce hospital readmissions (Becker et al. 2021; Manias et al. 2021; Ozavci, Bucknall, Jorm, et al. 2021; Tomlinson et al. 2020). Furthermore, effective communication is a critical factor of both patient‐centred care and transitions of care (Tobiano et al. 2019; Ozavci, Bucknall, Jorm, et al. 2021). In detailing the complexities influencing medication management across transitions of care, this study shows that an integrative approach needs to be used in interventions. Thus, the effectiveness of communication between older patients and healthcare professionals becomes more effective when it is supported within the context of structured, system‐level processes across the health system, which are geared towards consistent, clear and timely involvement of older patients and their families. Thus, interventions strengthen interpersonal communication by ensuring it is embedded into the workflow of healthcare professionals' activities within reliable, system‐level processes.

### 5.1 Strengths and Limitations

A strength of this study is that by conducting interviews across four sites of a major metropolitan hospital system, it was possible to include participants from diverse socioeconomic backgrounds. Furthermore, the large sample size and multiple ward types allowed for further diversity and rich data. Another strength of this study was the inclusion of the perspectives of patients, family members and healthcare professionals which allowed for the complexity of medication management to be explored from multiple lenses.

Through the use of dyad interviews, it was possible to gain rich data due to patients and family members reflecting on shared experiences and allowing for contextual insight. However, these dyadic interviews may also cause participants to be reserved and withhold or alter certain information due to the presence of their family member. Similarly, focus groups with healthcare professionals allowed for rich data and often prompted deeper insights; however, this interview format had some limitations. These included some participants dominating the conversation and the tendency for participants to just agree with what others have shared, rather than providing their own ideas. Furthermore, as these healthcare professionals were all colleagues, they may have withheld or altered information due to the presence of others. In an attempt to overcome this, interviewers made a conscious effort to capture perspectives of all participants by repeating the questions to each participant or asking them to expand on their answers and provide examples if they were in agreement with others.

Attempts were made to include persons who did not speak English, however limitations with interpreter availability and the tendency for persons who did not speak English to decline to participate, meant only one participant with no English ability was included in the study. Furthermore, the findings of this study may not align with the experiences and opinions of persons from rural or private hospitals.

### 5.2 Recommendations for Future Research

Future research should expand upon the identified communication shortcomings to design and test scalable strategies that are able to support enhanced communication through tailored information delivery, within the complex and time constrained healthcare system. Digital interventions could enable personalised plain‐language medication information in written, visual and audio formats, such as medication management applications for patient and family use, electronic discharge summaries, and enhanced teach‐back tools. Information delivery could be further personalised and automated through the use of artificial intelligence‐enabled systems, tailoring the information to individual patient and family needs, their health literacy levels, preferred language and cognitive ability. Such strategies have the potential to allow for more effective and efficient information delivery and improve shared decision making.

The design and development of these strategies should be conducted in collaboration with patients, family members and healthcare professionals, such as through co‐design workshops, to ensure that these strategies sufficiently address patient and family needs, without further burdening healthcare professionals. Evaluation of these strategies should focus on their impact on patient and family engagement in medication management and how this influences patient outcomes.

## 6 Conclusion

Although there was a desire for patients and family members to be engaged in medication management, communication breakdowns during transitions of care and barriers, such as complex language and understanding, limit their ability to engage. While there are multiple factors affecting this ability for engagement in medication management, strategies suggested by all participant groups to overcome these factors demonstrate the desire for approaches which encourage and empower patients and family. These proposed strategies, which support patients and family to participate in their medication management, have the potential to improve patient outcomes through addressing polypharmacy, reducing medication errors, and mitigating adverse events. These strategies can be incorporated within the context of structured, system‐level processes across the health system to facilitate maximum impact.

## Author Contributions

**Grace Marconi:** conceptualization, methodology, data curation, formal analysis, investigation, writing – original draft preparation, writing – review and editing. **Maneesh Prasad:** conceptualization, methodology, data curation, formal analysis, investigation, writing – original draft preparation, writing – review and editing. **Kerry Hwang:** conceptualization, methodology, data curation, formal analysis, investigation, writing – original draft preparation, writing – review and editing. **Caitlin Deery:** investigation, writing – review and editing. **Kelly Ottosen:** investigation, writing – review and editing. **Elizabeth Manias:** conceptualization, funding acquisition, methodology, project administration, supervision, writing – review and editing.

## Funding

This study was funded by the National Health and Medical Research Council (NHMRC), through an Investigator Grant (project no: 2025864).

## Ethics Statement

Ethical approval for this study was granted by Monash Health Human Research Ethics Committee. Ethics number: RES‐24‐0000‐199B.

## Conflicts of Interest

The authors declare no conflicts of interest.

## Supporting information


**Supporting Information S1:** Interview topic guide questions.

**Data S1:** Consolidated criteria for reporting qualitative studies (COREQ): 32‐item checklist.

## Data Availability

Data will be made available upon reasonable request to the corresponding author.
