# Supplementary material for: Perspectives About Older Adult and Family Member Engagement in Medication Management Across Transitions of Care
Source: J Clin Nurs. 2026 Jun 19;35(11):5016–28. doi: 10.1111/jocn.70405 (PMC13629292; doi:10.1111/jocn.70405)
Supplement: Supplementary file 2 [file JOCN-35-5016-s002.docx]

Supplementary Material

Supplementary Material 1. Interview topic guide questions

What questions can patients ask health professionals such as doctors, nurses or pharmacists, to gain understanding about medications that are prescribed?

What questions can family members ask health professionals such as doctors, nurses or pharmacists, to gain understanding about medications that are prescribed?

How can patients be more involved in making decisions about medications as patients move from one place to another?

How can family members be involved in making decisions about medication as patients move from one place to another?
